# Defining the Limits of Normal Conjunctival Fornix Anatomy in a Healthy South Asian Population

**DOI:** 10.1016/j.ophtha.2013.09.033

**Published:** 2014-02

**Authors:** Imran J. Khan, Abdul-Jabbar Ghauri, James Hodson, Matthew R. Edmunds, Paul Cottrell, Simon Evans, Geraint P. Williams, Saaeha Rauz

**Affiliations:** 1 Academic Unit of Ophthalmology, Centre for Translational Inflammation Research, University of Birmingham, Birmingham, United Kingdom; 2 Wolfson Computer Laboratory, Queen Elizabeth Hospital, Birmingham, United Kingdom; 3 Diamond Centre of Wales, Talbot Green, United Kingdom

## Abstract

**Purpose:**

Quantifying the extent of conjunctival fibrosis for documentation of progression in conjunctival scarring disease is a clinical challenge. Measurement of forniceal foreshortening facilitates monitoring of these disorders. This study aims (1) to define the limits of the normal human conjunctival fornices and how these alter with age and (2) to provide normative data for upper and lower fornix depths (FDs) and fornix intercanthal distance (FICD) within a healthy South Asian, racially distinct population.

**Design:**

Epidemiologic, cross-sectional study.

**Participants:**

A total of 240 subjects with national origins from South Asia, with no known ocular history and normal adnexal and conjunctival examination, aged 20 to 80 years.

**Methods:**

An FICD modification of a custom-designed fornix depth measurer (FDM) was validated and used for measurement of both lower and upper FDs together with FICDs in 480 healthy eyes with no ocular comorbidities. Data were analyzed using repeated-measures analysis of variance and presented as means with 95% confidence intervals (CIs).

**Main Outcome Measures:**

Mean lower and upper FDs and FICD for the entire cohort, stratified according to age decade and sex.

**Results:**

For this South Asian population, the overall upper and lower FDs were 15.3 mm (95% CI, 14.9–15.6) and 10.9 mm (95% CI, 10.7–11.1), respectively, with FICD defined as 32.9 mm (95% CI, 32.5–33.4) (upper) and 31.7 mm (95% CI, 31.3–32.1) (lower). With increasing age, a progressive reduction of all measured parameters (*P* < 0.001) was noted, with female subjects having significantly shallower fornices (upper FD, *P* < 0.001; lower FD, *P* < 0.001; upper FICD, *P* = 0.081; and lower FICD, *P* = 0.015).

**Conclusions:**

This is the first study to define the limits of normal upper FD and FICDs in any population group. Our study demonstrates sex variations and progressive conjunctival shrinkage with age. Although it provides important, objective data for normal forniceal anatomy, further study is recommended in other populations to confirm the generalizability of these data or to enable normal comparative datasets for the assessment of conjunctival scarring disorders among all anthropological groups.

Progressive cicatrizing conjunctivitis encompasses a group of conditions that are frequently associated with significant ocular morbidity and visual loss.^1^ Recurrent episodes of conjunctival inflammation lead to a compromised lacrimal functional unit characterized by progressive conjunctival subepithelial fibrosis and ocular surface failure with subsequent susceptibility to surface breakdown and blinding infections.^1^, ^2^ Objective measurement of forniceal foreshortening in conjunctival scarring disorders is necessary to confirm progression of disease and for accurate staging and monitoring of the disease process.^3^, ^4^, ^5^ Such disorders include ocular mucous membrane pemphigoid, Stevens–Johnson syndrome/toxic epidermal necrolysis, drug-induced cicatrizing conjunctivitis, and graft-versus-host disease.

Clinically, the fornix depth (FD) has been graded subjectively or semiobjectively by measuring the lower fornix with a slit-beam extending from the meibomian gland orifices or the limbus to the fornix boundary.^6^ These methods are limited because of alterations in the anatomic landmarks with both normal aging and ocular surface disease. A tool to measure the lower fornix, termed the “fornix depth measurer” (FDM), was originally described by Schwab and colleagues^7^ in 1992. More recently, FDMs have been introduced that have the additional capability of objectively quantifying the upper FD.^8^, ^9^ A limitation of all published FDMs to date is the inability to accurately measure horizontal forniceal obliteration by fibrosis.^9^ Documentation of this parameter is considered to be of critical importance when monitoring progressive conjunctival scarring^3^, ^4^, ^5^ but is frequently neglected in routine day-to-day clinical practice. Currently, no prototype instrument has been developed to enable accurate documentation of horizontal cicatrization.

Grading the presence of conjunctival fibrosis and progression of disease requires prior knowledge of normative values for the conjunctival fornices in the healthy population. This includes not only alterations in anatomic parameters that might occur with age or sex but also differences between anthropological races and ethnic groups. Only one study provides epidemiologic data measuring lower FD in a population according to age,^7^ but this study does not state the national origin or sex of the examined population group.

In our study, we use a novel modification of a previously validated FDM to evaluate upper and lower FD, together with upper and lower fornix intercanthal distance (FICD) in a healthy population with national origins from South Asia. We provide a normal dataset for upper and lower FD and FICD stratified according to age and sex, which will ultimately aid assessment and documentation of progressive conjunctival scarring within this population group.

## Methods

### Construction of a Combined Fornix Measurement Device

**Figure 1:**
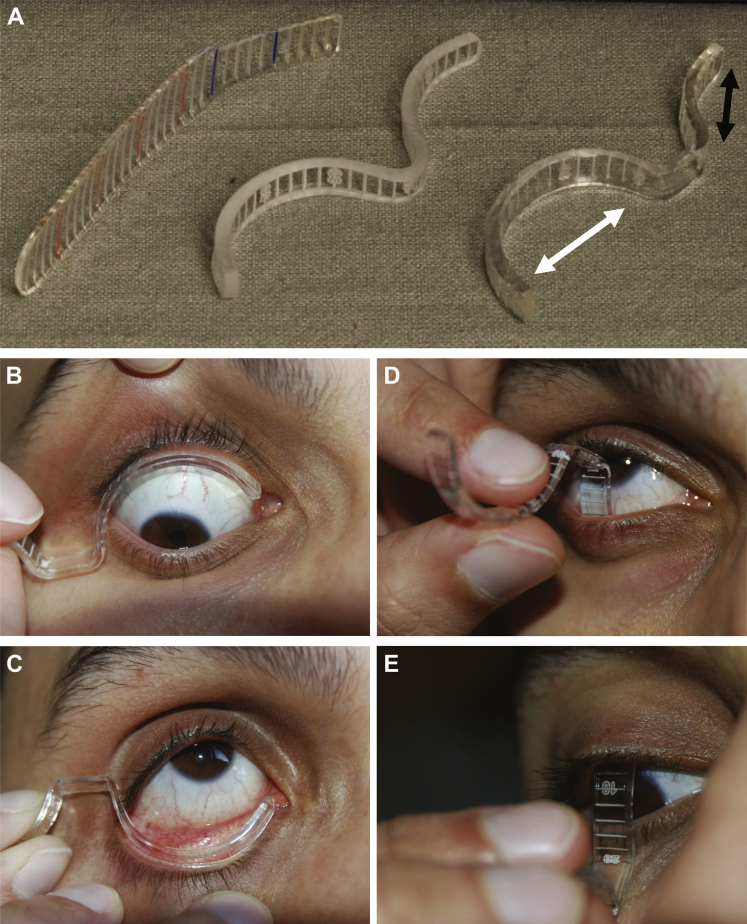
Construction and use of the conjunctival fornix measurer. **A,** Polymethylmethacrylate fornix measurer was constructed using industry-standard jewelry software and machinery. The original, validated polymethylmethacrylate fornix depth measurer (FDM),^6^ a semimolded but unpolished modified FDM, and the finished, polished prototype are shown (**A,***left* to *right*). The final prototype is a dual-ended biconcave design with engraved markings to a precision of 2 μm/step and increments expressed at 2-mm intervals. The longer end (*white arrow*) is used for the measurement of fornix intercanthal distance (FICD), and the shorter end (*black arrow*) is used to assess fornix depths (FDs). The fornix measurer was positioned after instillation of 1 drop of 0.4% oxybuprocaine hydrochloride. Subjects were asked to look in the opposite direction to the fornix being measured (up for the lower fornix, down for the upper fornix). The FICD was measured from the caruncle to the lateral canthus with the eyelid gently retracted **(B, C)**. For FD, the central conjunctival fornix was measured to the eyelid margin, defined as the posterior lip of the meibomian gland orifice **(D, E)**.

The modified fornix measurer was created using industry-standard jewelry computer software and cut to a precision of 2 μm/step (increments of 2 mm) (Fig 1A) molded to account for scleral curvature using measurements to fit scleral contact lenses (at the Birmingham and Midland Eye Centre, Birmingham, UK, and Moorfields Eye Hospital, London, UK) to enable FICD measurement.

### Subjects

Subjects aged 20 to 80 years with national origins from South Asia (Asian or Asian British as defined by the UK Census 2011, i.e., Indian, Pakistani, Bangladeshi, and Sri Lankan origin) were recruited into the study. All volunteers were residents of the United Kingdom. Subjects with a history of ocular surface disease, previous eyelid surgery, and any ocular disease requiring long-term topical treatment of any form (e.g., topical lubricants, intraocular pressure–lowering medication, topical steroids) were excluded. Each subject had an eyelid and ocular surface examination that included upper lid eversion to exclude subtarsal fibrosis. Subjects with evidence of ocular surface pathology including conjunctivochalasis were excluded.

### Validation of the Fornix Intercanthal Distance Modification

To validate the FICD modification of the FDM, masked, independent measurements of upper and lower FICD were undertaken by 2 observers (I.J.K., A.J.G.). Forty eyes of 20 volunteers with a median age of 41.5 years (range, 20–78 years) were evaluated. All FICD measurements were performed in triplicate, with the first of the 3 measurements used for interobserver comparison, and repeated in triplicate 1 hour later to estimate intraobserver error.

### Anthropological Study of the Ocular Fornix

To produce data for FD and FICD, sample size calculations indicated that 80 eyes per decade would be required to calculate means with 95% confidence interval (CI) widths of <0.8 mm. Forty healthy volunteers for each age decade from 20 to 80 years were recruited: 480 eyes of 240 volunteers in total.

Oxybuprocaine hydrochloride 0.4% was instilled to both upper and lower fornices before performing measurements. Subjects were instructed to look in the opposite direction to the placement of the fornix measurer (upgaze for lower fornix measurement, downgaze for upper fornix measurement) to prevent damage to the cornea and standardize the measurement technique (Fig 1B–E). The fornix measurer was sterilized as per local National Health Service Trust protocols for nondisposable applanation tonometer prisms, that is, immersion in 0.05% sodium dichloroisocyanurate for 5 minutes and rinsed with 0.9% sodium chloride between patients.

### Ethical Approval and Statistical Analysis

The study was conducted after written informed consent following an amendment to an ethically approved project (Birmingham East, North and Solihull Research Ethics Committee, IOSD 08H1206/165) and conformed to the tenets of the Declaration of Helsinki.

Intraobserver and interobserver variation of measurements taken with the modified fornix measurer were evaluated with Bland–Altman comparison of the differences in the measurements against mean measurements. The Bland–Altman comparison, the mean difference in observations, and the 95% limits of agreement were calculated using Excel for Windows (Microsoft Corp., Redmond, WA).

For the anthropological study, data were analyzed using repeated-measures analysis of variance to account for the correlation between the measurements on the left and right eyes of each volunteer. Age group and sex were the primary factors, with the interaction between these 2 variables also being tested. The analysis was performed using SPSS version 19.0 (IBM, Chicago, IL), with *P* values <0.05 taken as significant.

## Results

### Validation of Fornix Intercanthal Distance Measurements

Triplicate measurements of the upper FICD by each observer showed exact agreement of 70% (28/40) and 72.5% (29/40) of measurements by observers 1 and 2, respectively. One hundred percent of intraobserver observations were within 1 mm for observer 1, and 97.5% of intraobserver observations were within 1 mm for observer 2. Triplicate measurements of the lower FICD by each observer showed exact agreement of 52.5% (21/40) and 70% (28/40) of measurements within observers 1 and 2, respectively. One hundred percent of intraobserver observations were within 1 mm for both observers.

**Figure 2:**
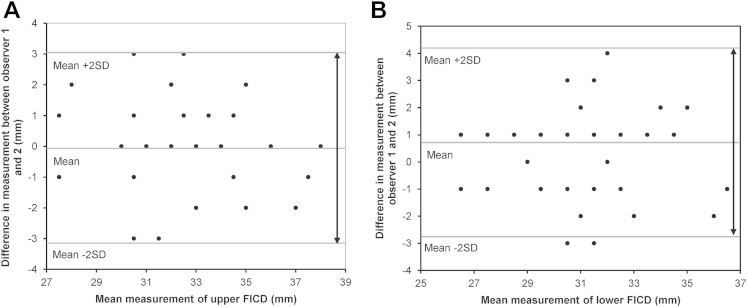
Bland–Altman plots evaluating interobserver variation of fornix intercanthal distance (FICD) measurements. Bland–Altman plots showing interobserver variation in upper **(A)** and lower **(B)** FICD. Because there are no defined limits for FICD, the calculations are in millimeters. The millimeter difference in assessment between observer 1 and 2 is plotted against mean millimeter measurement for each patient and the mean ± 2 standard deviations (SDs).

Interobserver variation of the upper FICD showed a mean difference in FICD measurement of 0.08 mm for observers 1 and 2, with 95% limits of agreement (±2 standard deviations) of −3 to +3 mm (Fig 2A), whereas interobserver variation of the lower FICD showed a mean difference of 0.65 mm, with 95% limits of agreement (±2 standard deviations) of −3 to +4 mm (Fig 2B). By using an allowance of 10% based on the acquired anthropological data (see next section), 98.75% (79/80) of FICD measurements were within 3 mm for both observers (3 mm <10% upper and lower FICD).

### Anthropological Data for the Ocular Fornices

There was no significant difference between right and left eyes for lower FD or FICD measurements (*P* = 0.498 and 0.160, respectively). Clinically insignificant differences were seen for upper FD (0.3 mm; *P* = 0.006; 95% CI, 0.1–0.5) and upper FICD (0.7 mm; *P* = 0.001; 95% CI, 0.5–0.9).

#### Upper and Lower Fornix Depths

The mean upper and lower FDs across our study population were 15.3 mm (95% CI, 14.9–15.6) and 10.9 mm (95% CI, 10.7–11.1), respectively.

**Figure 3:**
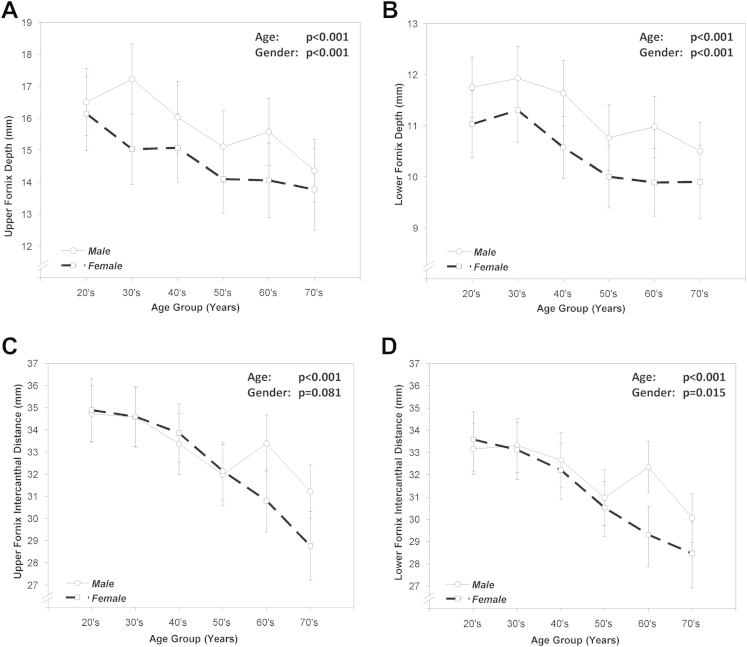
Estimated marginal means of fornix depths (FDs). Estimated marginal means of **(A)** upper and **(B)** lower FDs and **(C)** upper and lower **(D)** fornix intercanthal distance (FICD) per age group and separated by sex with 95% confidence intervals (CIs). A progressive decrease in both measurements for each of the parameters (FD, **A, B,** and FICDs, **C, D**) with age for male and female populations was observed (*P* < 0.001).

The mean upper FD was greater for male subjects compared with female subjects: 15.8 mm (95% CI, 15.4–16.2) versus 14.7 mm (95% CI, 14.2–15.2), respectively (*P* < 0.001). A similar pattern was seen for lower FD measurements (*P* < 0.001) (male subjects, 11.3 mm [95% CI, 11.0–11.5] vs. female subjects, 10.4 mm [95% CI, 10.2–10.7]). When stratified according to age, upper and lower FDs decreased with age, whereas female subjects had shallower fornices across all decades examined (*P* < 0.001) (Table 1; Fig 3A, B).

**Table 1:** Estimated Marginal Means of Upper and Lower Fornix Depths and Intercanthal Distances per Age Group and Separated by Sex

Age Decade	Sex (n)	Lower FD	Upper FD	Lower FICD	Upper FICD
20s	Female (36)	11.0 (7.0–15.1)	16.1 (10.6–23.2)	33.6 (27.7–41.4)	34.9 (28.6–43.6)
	Male (44)	11.8 (7.7–15.8)	16.5 (11.0–23.6)	33.2 (27.2–41.0)	34.7 (28.4–43.4)
30s	Female (40)	11.3 (7.3–15.3)	15.0 (9.5–22.1)	33.1 (27.2–41.0)	34.6 (28.3–43.3)
	Male (40)	11.9 (7.9–16.0)	17.2 (11.7–24.3)	33.3 (27.4–41.2)	34.6 (28.2–43.3)
40s	Female (42)	10.6 (6.6–14.6)	15.1 (9.5–22.2)	32.2 (26.3–40)	33.9 (27.5–42.5)
	Male (38)	11.6 (7.6–15.7)	16 (10.5–23.1)	32.7 (26.7–40.5)	33.4 (27.0–42.0)
50s	Female (36)	10.0 (6.0–14.0)	14.1 (8.5–21.2)	30.5 (24.6–38.4)	32.1 (25.8–40.8)
	Male (44)	10.8 (6.7–14.8)	15.1 (9.6–22.2)	31.0 (25.1–38.8)	32.0 (25.6–40.6)
60s	Female (36)	9.9 (5.9–13.9)	14.1 (8.5–21.1)	29.3 (23.4–37.1)	30.8 (24.5–39.5)
	Male (44)	11.0 (7.0–15.0)	15.6 (10.0–22.7)	32.3 (26.4–40.2)	33.4 (27.0–42.1)
70s	Female (30)	9.9 (5.9–13.9)	13.8 (8.2–20.9)	28.5 (22.5–36.3)	28.8 (22.4–37.4)
	Male (50)	10.5 (6.5–14.5)	14.4 (8.8–21.5)	30.1 (24.1–37.9)	31.2 (24.9–39.9)

FD = fornix depth; FICD = fornix intercanthal distance.

Data displayed as estimated marginal mean (95% confidence intervals); n, number of eyes per group.

#### Upper and Lower Fornix Intercanthal Distance

The mean upper and lower FICDs were 32.9 mm (95% CI, 32.5–33.4) and 31.7 mm (95% CI, 31.3–32.1), respectively. Both FICDs decreased with age (lower FICD, *P* = 0.015; upper FICD, *P* *<* 0.001) (Table 1; Fig 3B, C), but sex variation was detected only in the seventh and eighth age decades.

### Patient Comfort and Tolerance

As in our previous FDM study, the modified FDM was well tolerated by subjects, with only mild discomfort reported with upper fornix measurements in a few patients and no subjects reporting prolonged discomfort.^9^ There was no evidence of ocular surface mucosal epithelial abrasions.

## Discussion

This is the first study to define the normal limits of fornix anatomy in any population group. We provide a novel dataset that permits the clinician to objectively compare patients against normative values within a similar population. The generalizability of these data is yet to be explored.

Skull shapes fall into 3 broad categories originally described by Andres Retzius (1796–1860), who used dimensional analyses of the skull to generate the cephalic index terming brachycephalic (short skull, “East Asian Mongoloid”), mesocephalic (medium skull, “Negroid”), and dolichocephalic (long skull, “Caucasoid”) forms. Our South Asian population is captured within the dolichocephalic group. Because the shape of the dolichocephalic skull does not vary significantly across the incorporated ethnically and nationally diverse groups, which include White Europeans and South Asians, orbit morphology should not affect conjunctival FD. However, anecdotal evidence suggests that Indian subcontinent skulls tend to be more gracile and have milder brow ridges than European skulls with rounder orbits. Indian subcontinent skulls also appear to have lower nasal roots, more simple sutures, and mild prognathism and often appear to be narrower across the zygomatic region (Wilkinson C, personal communication, 2013). Further analysis is therefore required to define skull variations among ethnic subgroups within each skull anthropological hierarchy to determine whether these influence conjunctival fornix anatomy before accepting our data as generalizable to all skull forms and each subsidiary ethnic or national group.

The heterogeneous group of disorders that comprise progressive cicatrizing conjunctivitis can cause significant morbidity in the form of ocular discomfort, pain, and visual impairment. Recent evidence has shown that in a significant proportion of cases, subclinical inflammation leading to disease progression can occur, highlighting the need for more objective means of quantifying disease evolution.^10^ Clinical application of the fornix measurer and the normative dataset also may have roles in the assessment of other ocular diseases and their associated surgeries, such as thyroid-associated ophthalmopathy, ptosis, and giant fornix syndrome.

Other tools for measuring FD have been described in the literature, but these tools are restricted to measuring the lower fornix^7^ or the upper and lower fornix^8^, ^9^ or do not take account of the curvature of the globe.^8^ Our current prototype adds a method of assessing FICD together with FD, and our data are the first to provide normative values for these parameters. We demonstrated low intraobserver and interobserver variability in assessment of the FICD, facilitating repeatable and reproducible measurements of upper and lower FICD. However, reproducibility required a period of training, and even then, reproducibility was not as consistent as the upper and lower FD measurement. This indicates that further development of the prototype is required to improve ease of use and reduce the learning curve. Nevertheless, we provide the first FICD dataset to aid calculation of the percentage of horizontal obliteration of the fornices in conjunctival scarring disorders. Our results for mean lower FD support findings published by Schwab et al^7^ (anthropomorphic and ethnic data unknown) and those of the central upper FD (mean, 15.3 mm) provided by Kawakita et al^8^ (14.1 ± 2.5 mm superotemporal and superonasal upper FD, ethnically Chinese and Japanese-Asian subgroups). The differences observed in fornix size between sexes are likely to be attributed to differences in overall body size that is known to exist between male and female subjects within the same anthropological group.^11^

Of note, our data demonstrate that the upper and lower conjunctival fornix shrinks with age. This may be in part due to degenerative processes of elastic fibers comprising the extracellular matrix and is observed in various other tissues throughout the body.^12^ Alternatively, occult conjunctivochalasis may be the cause. Conjunctivochalasis is defined as redundant, loose, nonedematous inferior bulbar conjunctiva interposed between the globe and the lower eyelid associated with tearing and discomfort.^13^ Usually classified clinically, conjunctivochalasis may be associated with forniceal obliteration secondary to underlying inflammatory processes.^14^, ^15^, ^16^, ^17^ Although we excluded patients with any evidence of ocular surface pathology, early and asymptomatic conjunctivochalasis usually detected by imaging techniques, such as Fourier-domain optical coherence tomography,^18^ may not have been detected. Nevertheless, because of the high prevalence in an aging population, we speculate that conjunctivochalasis represents normal aging in the ocular surface mucosa impacted by an individual's genetic and exposomic makeup. Detailed exploration of how coexistence of conjunctivochalasis with age and how the molecular basis differs in different population cohorts provides scope for future study.

In conclusion, we present the largest epidemiologic study to date providing a normative dataset for a dolichocephalic, healthy, adult conjunctival fornix size in a South Asian population and demonstrate sex variability and age-related fornix shrinkage. By defining normal limits of anatomy, we provide a means for calculating the percentage of conjunctival shrinkage to facilitate detection and monitor progression of conjunctival scarring disorders, particularly those that are known to progress in a clinically quiescent eye.
